# Computational cardiology and risk stratification for sudden cardiac death: one of the grand challenges for cardiology in the 21st century

**DOI:** 10.1113/JP272015

**Published:** 2016-06-09

**Authors:** Adam P. Hill, Matthew D. Perry, Najah Abi‐Gerges, Jean‐Philippe Couderc, Bernard Fermini, Jules C. Hancox, Bjorn C. Knollmann, Gary R. Mirams, Jon Skinner, Wojciech Zareba, Jamie I. Vandenberg

**Affiliations:** ^1^ Victor Chang Cardiac Research Institute405 Liverpool StreetDarlinghurstNSW2010Australia; ^2^ St. Vincent's Clinical SchoolUniversity of New South WalesSydneyNSW2052Australia; ^3^ AnaBios Corporation3030 Bunker Hill St.San DiegoCA92109USA; ^4^ University of Rochester Medical CenterRochesterNY14642USA; ^5^ Global Safety PharmacologyPfizer IncMS8274‐1347 Eastern Point RoadGrotonCT06340USA; ^6^ School of Physiology, Pharmacology and NeuroscienceUniversity of BristolBristolUK; ^7^ Vanderbilt University School of Medicine1285 Medical Research Building IVNashvilleTennessee37232USA; ^8^ Computational Biology, Department of Computer ScienceUniversity of OxfordOxfordUnited Kingdom; ^9^ Cardiac Inherited Disease GroupStarship HospitalAucklandNew Zealand

## Abstract

Risk stratification in the context of sudden cardiac death has been acknowledged as one of the major challenges facing cardiology for the past four decades. In recent years, the advent of high performance computing has facilitated organ‐level simulation of the heart, meaning we can now examine the causes, mechanisms and impact of cardiac dysfunction *in silico*. As a result, computational cardiology, largely driven by the Physiome project, now stands at the threshold of clinical utility in regards to risk stratification and treatment of patients at risk of sudden cardiac death. In this white paper, we outline a roadmap of what needs to be done to make this translational step, using the relatively well‐developed case of acquired or drug‐induced long QT syndrome as an exemplar case.

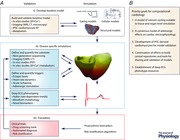

**Abbreviations**
aLQTS: acquired long QT syndrome
CICR: calcium‐induced calcium release
CiPA: comprehensive *in vitro* proarrhythmic assay
EC: excitation–contraction
ECG: electrocardiogram
EP: electrophysiology
HPC: high performance computing
HR: heart rate
ICD: implantable cardioverter defibrillator
IPSC: induced pluripotent stem cell
SCD: sudden cardiac death
SR: sarcoplasmic reticulum
TdP: torsades de pointes

## The challenge

Sudden cardiac death (SCD), which is most commonly caused by cardiac arrhythmias, accounts for ∼10% of all deaths in developed countries (de Vreede‐Swagemakers *et al*. 1997; Deo & Albert, 2012). Patients who suffer a SCD have some underlying cardiac pathology, even if they were unaware of it at the time. While implantable cardioverter defibrillators (ICDs) appear very effective at reducing the incidence of SCD, they remain expensive, and are not devoid of significant adverse effects such as inappropriate shocks, broken leads requiring invasive replacement, infections, and surgical complications, particularly in paediatric patients (Rosenqvist *et al*. 1998; Schimpf *et al*. 2003; Olde Nordkamp *et al*. 2015). Therefore, in order to optimise the use of ICDs, there is a critical need to correctly identify the patients who are at most risk and so most likely to derive benefit. At present our ability to identify these patients is poor and largely based on non‐specific associations such as the extent of contractile dysfunction in patients with heart failure (Stevenson & Epstein, 2003), or altered cardiac autonomic function (Nagahara *et al*. 2008). In the modern era of genomics‐inspired precision medicine, surely we can do better? In the last 20 years we have made great strides in understanding the molecular and cellular events underlying cardiac arrhythmias (Keating & Sanguinetti, 2001; George, 2013). The challenge now is to translate this knowledge into improved prediction and prevention of cardiac arrhythmias and SCD in patients.

Integrating the activity of the dozens of different types of ion channels and electrogenic transporters in multiple different cell types that underlie the electrical signals recorded on the electrocardiogram (ECG) is an extraordinarily complex task. Indeed, over 50 years ago Denis Noble, co‐founder of the Human Physiome project (Hunter *et al*. 2002; Hunter & Borg, 2003), recognised that it could only be achieved using high performance computing (HPC). Today's computers are many orders of magnitude more sophisticated and modelling of cardiac electrical activity is probably the most advanced of all the different components of the Human Physiome project. Even so, computational cardiology is not yet sufficiently advanced that it can be directly applied in the clinic. However, the establishment of a multitude of start‐up companies in this space suggests that we could be on the cusp of a revolution. Perhaps the strongest evidence to suggest that this may be the case is the Food and Drug Administration in the USA investing significant resources in the comprehensive *in vitro* proarrhythmia assay (CiPA) initiative. This new paradigm has *in silico* modelling as one of its core components for the pre‐clinical assessment of the proarrhythmic risk of all new drugs prior to clinical development (Sager *et al*. 2014; Fermini *et al*. 2016).

In this white paper, we will discuss the requirements for computational cardiology to become a reality in the context of predicting risk of cardiac arrhythmias. As a first step, we propose a roadmap for the development of such application and then discuss recent progress with the CiPA initiative and risk stratification for drug‐induced or acquired long QT syndrome (aLQTS) as an exemplar case.

## A proposed roadmap for computational cardiology

Arrhythmias are often discussed as falling into three groups: (1) triggered activities caused by early or delayed after depolarisations that may manifest as tachyarrhythmias; (2) altered automaticity resulting in either brady‐ or tachycardia; and (3) re‐entrant, where circus movement around areas of anatomical or functional block manifests as monomorphic/polymorphic ventricular tachycardia or fibrillation, depending on the stability and anchoring of the rotor (Antzelevitch & Burashnikov, 2011; Qu & Weiss, 2015). More recently, discussion has focused on the concepts of underlying substrates (that may be either structural *or* electrical in nature) that permit re‐entry and how they may interact with triggers in the genesis and maintenance of sustained arrhythmias (Kalin *et al*. 2010). The challenge in developing computational approaches to risk stratification for arrhythmias is to accurately define what we mean by substrates and triggers, and then to quantify these factors such that we can use them to generate mathematical models that can be validated against quantifiable outcomes. Lastly, and more importantly, the models then need to be able to make quantitative predictions that can be tested against the endpoint of clinical concern: proarrhythmia (see Abstract figure).

### Defining and quantifying substrates for arrhythmogenesis

Arrhythmias are emergent properties of intact tissue. Nevertheless, tissues are made up of cells and molecules and so quantifying tissue level properties will also require quantifying the genetic/molecular as well as cellular/intercellular components that make up tissues. For example, consider the scar tissue that forms after a myocardial infarct. At a tissue level, the extent of the scar tissue should be imaged as accurately as possible, as well as the relative distribution of fibroblasts *versus* extracellular matrix within the scar and border zone. Within these regions it is also important to understand the degree of remodelling of electrical and calcium handling properties, as well as the extent and spatial heterogeneity of sympathetic denervation (Li *et al*. 2004; Gardner *et al*. 2015). Ideally, one should also be aware of the genetic makeup of patients in order to comprehend potential impact on their baseline electrophysiological profiles. Adding further complexity, dynamic changes to the substrate such as variation in extracellular electrolytes, alterations in blood flow and supply of oxygen and nutrients secondary to acute episodes of ischaemia are important as they may explain, at least partly, why the same triggers can initiate arrhythmias on some occasions but not others. Finally, when thinking about dynamic changes it is also important to consider timescales. The above modifications are likely to occur over minutes to days. However, more rapid changes such as changes in heart rate (HR) can also alter how triggers will interact with the substrate.

### Defining and quantifying triggers for cardiac arrhythmias

It has long been recognised that an appropriately timed premature beat, i.e. a trigger delivered during the so‐called ‘vulnerable window’, has the propensity to initiate an arrhythmia (Mines, 1914). Indeed, the programmed application of premature, often multiple beats, forms the basis of most invasive electrophysiology (EP) studies aimed at determining the inducibility of arrhythmias (Doherty *et al*. 1983; Hummel *et al*. 1994). One challenge though is defining the conditions that determine a trigger in the setting of a dynamic substrate. For example, an ectopic beat delivered at a given coupling interval may not initiate an arrhythmia when the underlying HR is fast, but may do so when the underlying HR is slower. Furthermore, an ectopic beat of a given coupling interval and underlying HR may be more or less proarrhythmic depending on the prevailing autonomic tone. Fortuitously this is precisely where computational approaches can be most useful. In such complex scenarios, the very large number of combinations of variables may be prohibitive to test in wet lab experiments. However, with sufficient computational power hundreds and thousands of combinations of variables can be readily tested *in silico* and then only the critical variable combinations tested *in situ* or *in vivo*.

### Developing mathematical models

When it comes to quantifying each variable and incorporating them into mathematical models, another factor that we need to consider in the face of such a challenging and complex phenomenon, is how comprehensive do these models need to be – and to what extent can they be simplified? Bearing in mind the dictum that ‘all models are wrong but some are useful’ (Box, 1976) – we need to include enough information for models to be useful, but no more than is appropriate for the task, given the constraints of computational load as well as limitations in properly constraining the model. Fortunately, this is a challenge that has been embraced over many decades, starting with the pioneering work of Denis Noble (Noble, 1962). As a result, we now have a wealth of model frameworks from the single‐channel level, through to the whole‐heart to work with. Nevertheless, if we are going to reach the summit of developing models that have genuine clinical utility then understanding the limitations of each component of each model, and determining where simplification can and should (and indeed should not) be made, is always going to be critical. Inevitably, this will be an iterative process. It will be critical to use data that provide quantitative limits on substrates and triggers, to ensure that the models ultimately will produce predictions that are relevant to arrhythmias.

A particularly good example of an area in which model development and the assessment of the most appropriate level of model complexity merits close attention is calcium handling and excitation–contraction (EC) coupling. Calcium signalling is tightly coupled to the electrical properties of the heart, such that any disturbance to the electrical system will affect calcium signalling, and vice versa. Therefore, in understanding both the genesis and functional consequences of rhythm disturbances that can lead to SCD, this is a critical area of focus. There has been a huge amount of work exploring the molecular and cellular basis of calcium handling in isolated cardiac myocytes and its contribution to EC coupling (Bers, 2001, 2002). However, modelling calcium handling even at a single cell level is much more complex than describing the electrical properties of the cell. In the case of modelling intracellular calcium concentrations, both spatial gradients and microarchitecture of the subcellular compartment are potentially just as important as temporal changes, making this a much more computationally challenging problem. Over the years, modelling approaches to this phenomenon have evolved as our knowledge of intracellular calcium handling has grown. Starting with models where intracellular calcium exists purely as a function of calcium influx through the plasma membrane (Beeler & Reuter, 1977), we have seen the addition of intracellular compartments (the sarcoplasmic reticulum) that sequester and release calcium (DiFrancesco & Noble, 1985), linking of these compartments to membrane calcium currents to describe calcium‐induced calcium release (CICR) (Luo & Rudy, 1994) and addition of diffusion restricted sub‐compartments of the cytoplasm approximating the environment of the junctional SR–T‐tubule interface (Jafri *et al*. 1998). The modelling of calcium homeostasis has been subsequently refined to include more accurate representations of calcium removal mechanisms and the dependence of CICR on SR load (Shannon *et al*. 2004; Grandi *et al*. 2010), the kinetics of phosphorylation of calcium handling proteins by CaMKII (Iribe *et al*. 2006), and updates of L‐type calcium current and Na^+^–Ca^2+^ exchanger based on data from human ventricular tissue (O'hara *et al*. 2011). In all of these models, calcium concentration is spatially homogenised in each compartment, i.e. takes the same value everywhere in the compartment at a given time. More recently, microdomain models have been developed that include structural as well as molecular descriptions of the dyadic cleft to varying degrees of accuracy (Schendel & Falcke, 2010; Schendel *et al*. 2011; Cannell *et al*. 2013; Stern *et al*. 2013), that can be scaled up to describe whole cytoplasmic calcium concentrations (Higgins *et al*. 2007; Restrepo *et al*. 2008; Rovetti *et al*. 2010; Gaur & Rudy, 2011; Nivala *et al*. 2015; Vierheller *et al*. 2015). This reductionist approach necessitates the simulation of tens of thousands of dyads per cell, requiring significant computational times between ten minutes to many hours for a single beat of an individual cell, depending on the level of detail included (Nivala *et al*. 2012; Vierheller *et al*. 2015). As a result, while this certainly represents a gold standard for simulation of calcium dynamics in single cells, it presents a problem for consideration of larger scale tissue or organ level simulations that will likely require implementation of multiscale computational strategies. This example therefore embodies perfectly the conundrum described above, of selecting models of appropriate level of complexity for the task at hand. It also highlights an area where particular focus should be applied in advancing numerical techniques or taking advantage of new, more powerful parallel computing hardware (Tuan *et al*. 2011; Chai *et al*. 2013), such that more complete descriptions of calcium handling can be incorporated into tissue level simulations.

### Model validation

Once we have established the required models, thorough validation is critical, and this must use datasets independent of those used to generate the models in the first place. At the cellular level, one area of growth that will no doubt play a central role in validating cellular models is the use of cardiomyocytes derived from human induced pluripotent stem cells (IPSC) (Takahashi & Yamanaka, 2006; Narsinh *et al*. 2011). IPSC derived cardiomyocytes from patients with normal as well as diseased hearts present a platform for studying cardiac diseases (Carvajal‐Vergara *et al*. 2010; Moretti *et al*. 2010) or drug effects (Sinnecker *et al*. 2014) in a more faithful biological context. Critically, these ‘hearts in a dish’ maintain many intrinsic disease characteristics and genetic background of the patient, and so represent a powerful tool to study the mechanisms of disease, or the actions of drugs, without the ethical and practical implications of collecting human cardiac tissue. However, this technology is still in its relative infancy. In particular, issues exist around the relative immaturity of these cells and how well they reflect the function and morphology of the adult ventricular myocyte in relation to electrophysiology, calcium handling and responses to adrenergic stimulation for example (Laflamme & Murry, 2011). It is certainly true that efforts are already well underway to tackle this issue. Aside from long term culture on the order of months to years (Otsuji *et al*. 2010), more recent efforts have focused on efforts to mimic more closely the environment of the developing heart and drive these cells to a more adult phenotype such as 3D culture and mechanical stimulation (Nunes *et al*. 2013; Mihic *et al*. 2014), electrical stimulation (Eng *et al*. 2016) and co‐culture with non‐cardiomyocyte cells (Kim *et al*. 2010; Saini *et al*. 2015). Culturing of IPSC derived cardiomyocytes on microgrooved substrates has proved particularly effective in promoting more adult‐like cellular morphology as well sarcomeric organisation, that in turn is important for defining the subcellular spatial restrictions necessary for normal calcium homeostasis. Under these conditions, cells showed calcium transients that more faithfully reflected those observed in adult myocytes (Rao *et al*. 2013). These approaches have therefore shown a great deal of promise in promoting both electrical and morphological maturity, yet there is no doubt that these cells are still more fetal than adult in nature. Despite this, the technology is rapidly being embraced – most notably as part of the CiPA initiative (Sager *et al*. 2014). As such, it is critical that issues surrounding maturation of IPSC derived cardiomyocytes are tackled such that this rapidly emerging field can play a central role in the validation and construction of cellular electrophysiological models.

Ultimately though, sudden cardiac death represents the final common endpoint of abnormal electrical signalling in the organ, i.e. is not solely a cellular phenomenon. First and foremost therefore, any computational model must be able to reproduce, at a quantitative level, the electrical signals recorded from patients. In this regard, we foresee a very important role for deep ECG phenotyping and specifically for high‐resolution analysis of Holter ECGs that provide information on how the electrical signalling in the heart responds to a wide range of physiological stimuli. Until recently, the analysis of Holter ECGs has been limited (due to the relatively large volumes of data acquired compared to the computational power available to analyse them). Recent increases in computer processing power and storage capacity have largely obviated this problem, but there is still a lot that can and should be done to develop better software packages for analysing multi‐lead Holter ECG datasets, which will be very useful for validating computational models. This again should be seen as an iterative process and indeed one can envisage that this process will lead to identification of better biomarkers of cardiac electrical activity that in themselves may provide better means for stratifying risk of SCD.

## Drug‐induced arrhythmias: a case study for *in silico* risk prediction

In the past 15 years, a range of structurally unrelated non‐cardiovascular drugs have been withdrawn from the market due to adverse effects on cardiac repolarisation and risk of heart rhythm disturbances – so called acquired or drug‐induced long QT syndrome (aLQTS) (Wood & Roden, 2004). These drugs include antihistamines, antibiotics, antipsychotics and most recently the analgesic propoxyphene, which was prescribed to an estimated 10 million patients in the US at the time of its withdrawal in 2010. The aLQTS is characterised by delayed repolarisation, prolongation of the QT interval on the surface electrocardiogram (ECG) and a markedly increased risk of a potentially lethal ventricular arrhythmia named torsades de pointes (TdP) (Wood & Roden, 2004; Kannankeril *et al*. 2010). aLQTS can be caused by drugs that block any of the ionic currents that contribute to repolarisation of the heart. In practice, however, the vast majority of drugs that can cause aLQTS do so by inhibiting hERG channels (Perrin *et al*. 2008 *b*). This observation led to the current suite of guidelines relating to cardiac safety that were issued in 2005: recommending that, at a minimum, all compounds are subject to an *in vitro* evaluation of hERG block together with assessment of *in vivo* QT interval prolongation in an appropriate animal model (ICH S7B) and an assessment of QT prolongation in humans (ICH E14) (Food and Drug Administration, HHS, 2005 *a*,*b*).

These guidelines have several important shortcomings. First amongst them is that while the tests are very sensitive, and have been successful in eliminating dangerous drugs, they are believed to be insufficiently specific. Neither hERG block nor QT prolongation are good predictors of risk of TdP (Sager *et al*. 2014). There is also growing concern that this approach has led to the premature termination of the development of drugs based on their hERG affinity, rather than propensity to actually cause arrhythmia. In the context of the roadmap outlined in the Abstract figure, potency of hERG block and QT prolongation do not provide sufficiently accurate markers of the proarrhythmogenic substrate to be useful for quantifying risk. These shortcomings are acknowledged by the regulatory agencies, which has led to the establishment of CiPA. This initiative is set to change the regulatory guidelines around cardiac safety testing to a multi‐ion channel, rather than hERG‐centric approach, that is driven by *in silico* risk prediction (Sager *et al*. 2014; Fermini *et al*. 2016). This new approach will take into account detailed characterisation of molecular pharmacology including ion channel–drug interaction kinetics as well as multichannel block profiles with the idea that simulations of cardiac electrophysiology based on this mechanistic insight will better identify proarrhythmic drugs.

However, while CiPA focused on the use of single cell modelling for prediction of proarrhythmic propensity, in reality the genesis of arrhythmia in the intact heart involves so much more than solely the molecular interaction of ligand and target. Drugs that block the hERG channel both delay repolarisation and increase the dispersion of repolarisation across the heart (Kannankeril *et al*. 2010). The delayed repolarisation is considered to increase the probability of reactivation of L‐type calcium channels leading to early after‐depolarisations (EADs) that could act as the ‘trigger’ for arrhythmia (January & Riddle, 1989). Additionally, the increased transmural repolarisation heterogeneity produces regions of functional block and slow conduction that provide an intramural substrate for re‐entry (Akar & Rosenbaum, 2003). Although we understand in principle the tissue level mechanisms of arrhythmogenesis in aLQTS, the link between hERG block and the emergent arrhythmia is complex. However, the processes that need to be in place to interrogate this *in silico* are relatively well established, meaning the aLQTS example is an ideal illustration of the computational risk prediction pipeline outlined in the Abstract figure. The specifics of model development, defining and quantifying substrates and identification of novel risk biomarkers from multiscale models in relation to aLQTS are discussed below.

### Development and optimisation of models for *in silico* risk prediction in aLQTS

An important step in the pursuit of effective *in silico* risk prediction is the selection and optimisation of the molecular and cellular models used for studying the action of pharmaceutical compounds. At the cellular scale, we need to reach a consensus on an appropriate action potential model. This is a critical step given the dramatic range in action potential morphology that exists between published models (Cooper *et al*. 2016) as well as the intrinsic properties of the models, such as forward/reverse rate dependence, that must be considered in understanding how individual models respond to external perturbations (Cummins *et al*. 2014). In either case, variation in maximal current densities (maximal ‘conductances’ in the case of ion channels) is a major source of discrepancy between predictions from different models. Unfortunately, most of these conductances are not easy to measure directly, particularly from healthy human cardiomyocytes, and have to be inferred from action potential recordings and cell‐level behaviour. While some recent models of the ventricular action potential have been based on data from healthy human hearts (O'hara *et al*. 2011), there is still work to be done in tuning these models for the range of physiological stressors that are critical in arrhythmogenesis such as their responses to adrenergic stimulation. In this regard, the use of IPSC derived cardiomyocytes (see Model validation section, above) will no doubt contribute to providing the datasets necessary for this ongoing model development and validation.

Equally important in determining model predictions is the choice of model for each ion current, specifically concerning channel gating kinetics in control conditions. Here, most models use Hodgkin–Huxley or Markov model structures for the ion channel states and transitions. This is not a simple task. For example, there are at least seven different published model structures for the *I*_Kr_ current alone (Noble *et al*. 1998; Lu *et al*. 2001; Piper *et al*. 2003; Adeniran *et al*. 2011; Bett *et al*. 2011; Di Veroli *et al*. 2013 *b*). When we consider different parameterisations of these model structures (different transition rates and their voltage dependence), there are over thirty published model variants for *I*_Kr_. There is therefore uncertainty in both the model structure/equations and also the parameters which should be used (see Mirams *et al*. (2016): another white paper in this issue). Quantifying this uncertainty will be an important goal in the coming years, and will be necessary to place an appropriate level of trust in model predictions (Johnstone *et al*. 2015). As part of this effort, we should quantify which experiments provide information on parameters and structures: a whole sub‐field of statistics/control theory called ‘optimal experimental design’ is dedicated to this task, but only a few steps have been taken so far in applying this to cardiac modelling (Dokos & Lovell, 2004; Fink & Noble, 2009; Groenendaal *et al*. 2015). It is also worth noting that a perfect fit to training data is not a sign that the model will be predictive in future, particularly if multiple model structures and/or parameter sets provide an equally good fit (Fink *et al*. 2011). Models should always be evaluated in the context of predicting an outcome that they were not trained to replicate.

Each of these factors, the selection of cellular and molecular models, have important bearing in terms of evaluating predictions of drug‐induced proarrhythmic risk, such as those envisaged by CiPA, where *in silico* predictions are currently based mostly on fits to standardised *in vitro* datasets. In this regard, a gold‐standard model for use in computational evaluation of proarrhythmic risk may be the largest gap in our knowledge. Careful choice and further calibration and validation of ion current and action potential models remains one of the fundamental challenges for computational physiology in the coming years that is necessary to predict proarrhythmia associated with acquired LQTS more accurately. To ensure transparency and engender confidence in such computational approaches, we need to publish: (i) training data; (ii) calibration/fitting and selection algorithms that give rise to the final model; and (iii) validation data and performance metrics. This approach is in line with the general trend within science of moving towards ‘open data’ with a view to ensuring reproducibility, especially in computational science. In this regard, platforms such as Zenodo (hosted at CERN), datahub.org and researchcompendia.org provide the infrastructure for publishing and sharing of scientific data and models while many discipline specific repositories have also been built in recent years (see e.g. NIH Data repositories: https://www.nlm.nih.gov/NIHbmic/nih_data_sharing_repositories.html). Of particular relevance to the Physiome community, the CellML effort allows us to share model equations and parameters easily and provides a forum for model curation to ensure consistency of implementation between groups (Lloyd *et al*. 2008), while the recently established Cardiac Electrophysiology Web Lab (Cooper *et al*. 2016) allows functional curation of CellML encoded models, letting users compare and contrast how different models behave in response to a suite of virtual protocols. Perhaps the research field in which the discussion around data/model sharing is most advanced is genomics. The Broad Institute recently published white paper discussing the international standards and computational needs to share and integrate data in a secure, controlled and interpretable manner (Alliance, 2014). This can serve as a worthy template for the action that should be taken by the Physiome community which should be encouraged and indeed mandated by funding agencies and consortium heads to ensure reproducibility in our discipline.

### Defining and quantifying substrates in aLQTS

In the aLQTS the primary substrate is abnormal electrical function precipitated by the block of cardiac ion channels (primarily hERG) as a side‐effect of both cardiac and non‐cardiac drugs. Specifically, the disruption of the repolarisation reserve is thought to amplify transmural spatial dispersion of repolarisation, establishing voltage gradients that can act as a substrate for re‐entry (Antzelevitch, 2007), while other factors such as temporal dispersion of repolarisation (i.e. beat to beat instability) are also thought to contribute (Frommeyer & Eckardt, 2015). The challenge of quantifying the substrate therefore lies in accurately defining and modelling the pharmacology of drug–channel interactions in the context of the cardiac action potential. To do this requires that we revisit the question of what degree of complexity these models must encapsulate to make *in silico* prediction of their proarrhythmic propensity a reality?

#### Measuring and modelling hERG block

Whilst it is certainly true that potency of block is not a good indication of proarrhythmia (Redfern *et al*. 2003; Di Veroli *et al*. 2013 *a*), a key outstanding question in determining the extent to which it is necessary to develop biophysically accurate models of drug binding is: are the kinetics of drug interaction with hERG, and indeed other channels, important in determining proarrhythmic risk? In support of this concept, Di Veroli *et al*. proposed that kinetics, rather than multichannel block profiles, might underlay the difference in proarrhythmic propensity between the drugs verapamil and bepridil (Di Veroli *et al*. 2013 *a*). More recently, Lee *et al*. demonstrated *in silico* how the kinetics of drug binding can modify both the degree of prolongation observed with an IC_50_ concentration of drug well as the Action Potential (AP) morphology, in terms of the degree of triangulation for example (Lee *et al*. 2015), that reflect likely differences in the associated risk profile (Hondeghem & Carlsson, 2001). This study, however, was completely theoretical, and it remains to be seen where real drugs fall into this theoretical landscape, and whether the range of kinetics observed *in vitro* allows for these complex dependencies.

When generating models of the kinetics of drug binding to ion channels, many of the same issues that arise in relation to modelling ion channel function also come to light. Multiple published models exist whose structures vary according to considerations such as to which states does a compound bind/unbind (Di Veroli *et al*. 2013 *a*); is binding voltage dependent (Moreno *et al*. 2011); do drugs get trapped behind a closed activation gate (Mitcheson *et al*. 2000); or does drug binding alter the intrinsic gating behaviour of the channel (Lee *et al*. 2015)? The key to addressing these issues *in silico* lies with accurately measuring and modelling the kinetics of drug binding to hERG (as well as other channels). Much of the complexity in achieving this stems from the fact that drugs can differentially bind to the open and inactive states of the hERG channel (Suessbrich *et al*. 1997; Walker *et al*. 1999; Weerapura *et al*. 2002; Perrin *et al*. 2008 *a*). Most studies assessing binding kinetics depend on complex voltage protocols that result in cycling of the channel between open and inactive states in a voltage dependent manner (Vandenberg *et al*. 2012). As a result, it is difficult to confidently deconvolute drug binding kinetics from voltage dependent channel gating. One way to tackle this problem is to directly monitor the drug–channel interaction at a single voltage (Milnes *et al*. 2010; Hill *et al*. 2014). Using similar approaches with other drugs presents us with the opportunity to accurately model the mechanism by which drugs block the hERG channel, and interrogate in detail questions relating to drug binding kinetics. For example, how do the kinetics of binding and unbinding vary with temperature? This is a question that is critical to answer if we want to extrapolate from data acquired in high throughout systems at room temperature to physiological temperatures *in silico*. How do the kinetic properties of the drug correlate with the observance of proarrhythmic markers such as triangulation, reverse use dependence, instability and dispersion (TRIaD criteria) and indeed do any specific kinetic properties correlate with risk?

With a view to answering these questions, a central tenet of CiPA is to include kinetics of block, rather than just potency, in their *in silico* models to estimate risk. However, CiPA is bound by practicalities of industrial screening in the extent to which kinetics can be measured. For example, most high‐throughput patch clamp systems are not able to switch perfusion solutions fast enough to allow direct, accurate measurement of drug binding and unbinding. Furthermore, the extent to which the kinetics of drug binding are actually important in determining risk is not yet fully understood. As a result, first‐pass measures of drug‐binding kinetics deemed appropriate for CiPA may not necessarily equate to the more academically attractive endpoint of fully characterising the kinetics of drug binding in order to constrain mechanistically accurate models of the interaction. However, it is clear the most effective and efficient approach to tackle this problem in the coming years is to leverage the resources and expertise available across the range of stakeholders from academia, the pharmaceutical industry and regulatory bodies to ensure that we have the right predictive models that will benefit patients.

#### Modelling multichannel block

Alongside hERG‐specific drug interactions, it is increasingly clear that a more complete pharmacological profile of individual compounds needs to be considered in order to properly assign risk (Mirams *et al*. 2011; Kramer *et al*. 2013), and therefore needs to be described *in silico* as we work towards holistic models of drug effects. Even a small degree of mixed channel block can shorten or prolong the QT interval by a few milliseconds, enough to be measured in a clinical ECG study. However, it is not known what (if any) risk is associated with these changes. It is therefore important to understand whether small degrees of block of multiple cardiac ion channels can combine to give rise to increased proarrhythmia, or alternatively perhaps confer some safety margin by compensating for block that would ordinarily be proarrhythmic. Such effects are certainly observed in the clinic, with drugs such as verapamil and amiodarone for example, whose multichannel pharmacology, especially block of cardiac calcium channels, appear to significantly abrogate the risk profile that would be associated with their block of hERG. Mathematical models offer an intuitive and biophysically based way to combine the effects of multiple ion channel block in order to tackle this question. *In silico* approaches based on concentration–effect data derived from high‐throughput drug screens on multiple ion channels have been examined in this regard and have shown good qualitative and quantitative agreement with experimental results from rabbit ventricular wedge preparations (Beattie *et al*. 2013) but less good prediction of results from thorough QT studies (Mirams *et al*. 2014), suggesting there is still work to be done in this area. A limitation of these studies is that simple concentration–effect data do not take into account the full complexity of drug–channel interactions, such as descriptions of binding and unbinding kinetics as described above for hERG, meaning this is an obvious area to address in seeking to improve *in silico* models of multichannel block. It should be noted, however, that development of Markov‐type descriptions of channel gating and pharmacology for multiple ion channels represents a major undertaking, so once again warrants careful consideration of the depth of characterisation that is deemed necessary.

A second aspect to consider in relation to multichannel block pertains to polypharmacy. Many, if not most, adverse proarrhythmic effects are seen in patients who are taking multiple drugs (‘torsade coadministration’ has > 16,700 hits on Google Scholar as of October 2015, see e.g. (Chézalviel‐Guilbert *et al*. 1995; Atar *et al*. 2003; Kounas *et al*. 2005; Kaźmierczak *et al*. 2007). With the correct application of *in silico* modelling of multichannel pharmacology, predictions could be used to avoid co‐administration of particularly dangerous compounds. This advance will require risk information to be disseminated to the doctors in the clinic differently: risk is no longer associated with the ‘black box’ for a particular drug, but a new ‘black box’ would be generated for the patient's particular prescription. However, in this scenario of combinations of compounds, each with multi‐target pharmacology, it is likely that historically trained statistical models will not be capable of accurate predictions – there is quickly a combinatorial explosion with many risk factors; so no training procedure will be able to assemble enough cases to cover ‘all the options’ we may wish to consider. For this reason, we consider that biophysically based mechanistic models will continue to have a larger predictive advantage over purely statistical models in future.

### Patient specific factors influencing the substrate in aLQTS

The major focus of current regulatory guidelines is the identification of compounds that carry risk of TdP. However, even in the context of ‘dangerous drugs’, the incidence of TdP is relatively low. For example, the incidence of TdP in randomised trials of dofetilde is reported to be 0.8–3.3% (Torp‐Pedersen *et al*. 1999; Singh *et al*. 2000). This raises the question of what makes these subsets of patients vulnerable to drug‐induced TdP? If we were able to tackle this problem, it may soon be plausible to move risk stratification from the pill to the patient, taking into account the sub‐groups that a patient falls into, in terms of age, sex, genetic background and disease states. All of these are well‐recognised risk factors for proarrhythmia (Zeltser *et al*. 2003; Frommeyer & Eckardt, 2015), and are known to influence the electrophysiology of the cell in ways that can be encapsulated in mathematical model parameters (Yang & Clancy, 2012).

#### Genetic background

The concept of ‘repolarisation reserve’ has provided the conceptual framework to understand why a person's genetic background may alter their response to QT prolonging drugs (Roden, 2006). The central tenet of this concept is that normal repolarisation is a function of several redundant mechanisms, where the overall repolarisation potential is determined by the level of expression and function of multiple cardiac ion channels, which in turn are coded for by an individual's genetic background. In this scenario, the effect of QT prolonging drugs occurs in the context of different electrical environments from person to person, meaning the level of risk is specific to the individual.

Our ability to characterise this electrical background, at least at the genetic level, has increased exponentially with the advent of next generation sequencing. In this regard, the early tranche of studies identified both cardiac (Weeke *et al*. 2014) and non‐cardiac (Jamshidi *et al*. 2012) genes involved in risk determination in aLQTS. In this time of precision medicine (Collins & Varmus, 2015), *in silico* approaches hold the promise of (1) allowing us to interpret this wealth of available data; (2) quantitatively analyse the effect of variable genetic backgrounds; (3) identify potentially protective and/or risky backgrounds, and most importantly (4) make predictions about how an individual or group of individuals may respond to a QT prolonging drug. Early efforts to tackle these questions have involved using multivariate analysis to examine how variable genetic background altered action potential duration (APD) prolongation in response to *I*_Kr_ block (Sarkar & Sobie, 2011), and provide an excellent platform for further *in silico* study of this area. At the cellular level this approach could be extended to better proarrhythmic markers such as triangulation or early after depolarisations (EADs), while simulation of organ‐level effects will allow us to take into account tissue properties relevant to proarrhythmia such as transmural dispersion (Akar & Rosenbaum, 2003), alternative ECG markers associated with risk (discussed below) and more complex rate dependent effects. In this regard, the potential to extend sensitivity analysis to the organ level now exists (Sadrieh *et al*. 2014), and presents an opportunity to tackle this problem in the coming years.

#### Tissue structure

A second factor to consider in identifying patients at risk is the tissue structure of the myocardium in each individual. A confounding effect in the current approach is that early phase clinical trials are carried out in cohorts of healthy individuals, and consequently do not take into account potential patient specific co‐morbidities such as tissue structure alterations that can influence the level of arrhythmia risk associated with a drug. The patients at greatest risk of cardiac adverse effects are likely to be those with pre‐existing heart disease and associated structural remodelling. For example, the patchy fibrosis characteristic of ageing, hypertensive heart disease and heart failure gives rise to structural discontinuities across a range of scales that can contribute to initiation of arrhythmia (Tanaka *et al*. 2007; Tusscher *et al*. 2007). Furthermore, the more compact fibrosis of healed myocardial infarcts can also provide a focused substrate for circus re‐entry (Jalife, 2000). It is likely that such structural abnormalities could help explain the variability in risk profiles observed with drugs that prolong the QT interval. This concept is supported by early clinical trials of antiarrhythmic drugs such as the Cardiac Arrhythmia Suppression Trial (CAST) that was designed to test the hypothesis that suppression of premature ventricular complexes with class I antiarrhythmic agents would reduce mortality after a myocardial infarction, but instead found that the tested drugs actually decreased survival (The Cardiac Arrhythmia Suppression Trial (CAST) Investigators, 1989; Echt *et al*. 1991).

From the perspective of computational physiology, multiple *in silico* approaches have already demonstrated the role of structural discontinuity in arrhythmogenesis (Caldwell *et al*. 2009; Engelman *et al*. 2010; Rutherford *et al*. 2012). Specifically, it has been shown that patchy fibrosis can give rise to re‐entrant activation as well as dynamic functional substrates that maintain re‐entrant circuits (Engelman *et al*. 2010). It is a short conceptual leap to suppose that these structurally defined substrates and triggers will amplify the specific proarrhythmic properties of drugs in aLQTS. Of particular interest in this regard will be an evaluation of rate dependent effects. For example, drugs that interact with the hERG channel with different kinetics and/or state dependence may well differentially affect the rate dependent instability that arises from structural defects such as fibrosis. A further factor to consider regarding the role of structural alteration in modifying risk is that the repolarisation heterogeneity, which is thought to contribute to initiation of arrhythmias in aLQTS, is amplified by structural remodelling (Akar & Rosenbaum, 2003). In the structurally normal heart, this heterogeneous repolarisation dispersion is modulated by electrotonic coupling (Nattel *et al*. 2011) that one could argue is reduced in structural heart disease, providing further amplification of proarrhythmic effects. Such problems, which are likely to include identifying precise mechanistic features of drug binding that contribute to structural‐dependent risk modification, are very difficult to tackle either *in vivo* or *in vitro*. However, *in silico* approaches offer the flexibility to quantitatively address these issues.

To achieve this, however, in addition to appropriate molecular models of drug–channel interaction, will also require high resolution structural imaging to provide accurate and detailed meshes for *in silico* approaches. Over the years imaging approaches have revealed detailed laminar architecture of the heart including collagen organisation and myofibre orientation that are critical in defining electrical propagation. Similar approaches have also been extended to imaging of infarct and peri‐infarct zones in animal models allowing *in silico* evaluation of how electrical and structural disruptions interact to precipitate potentially arrhythmic phenotypes (Rutherford *et al*. 2012; Deng *et al*. 2015). A critical area for development in this field, as we work towards clinical integration, will be the continued growth of imaged‐based analysis in human subjects. Imaged‐based models, that include details of structural discontinuities exist for both human atria (Zhao *et al*. 2013; McDowell *et al*. 2015) and ventricles (Wallman *et al*. 2014; Ukwatta *et al*. 2015), while recent developments have focused on techniques and pipelines for extracting useful structural meshes of human hearts and infarct scars from standard medical imaging approaches (Crozier *et al*. 2015; Ukwatta *et al*. 2015). These pipelines are steps toward structure‐based personalisation of *in silico* approaches that will, over time, also establish populations of cardiac structures. In the context of aLQTS, for example, this will allow us to consider more broadly how electrical disturbances that occur as adverse effects of drugs are influenced by the range of structural discontinuities present in human subjects, to provide a more nuanced consideration of risk stratification in this area.

### *In silico* driven risk stratification for aLQTS

Tackling the range of issues outlined above will provide the necessary inputs to facilitate organ‐level simulations aimed at identifying ECG biomarkers that are more predictive of TdP than the current standard of using the QT interval. Resolving this problem *in silico* embodies the motivation of the Physiome project. Many of the issues faced in risk prediction in aLQTS are related to the fact that it is almost impossible to predict the path from molecular event – drug binding to an ion channel in this case – to emergent properties of the cardiac electrical system that might manifest on the ECG as a useful biomarker for proarrhythmic risk. The ultimate goal for *in silico* risk prediction is therefore to incorporate the factors discussed above, including details of molecular interactions, cellular biophysics, tissue structure and geometry of the heart, into multiscale, organ level simulations that can predict these emergent outputs, which can then be correlated with the degree of risk associated with known compounds, validated against clinical ECG data, and used for future risk prediction.

#### Organ level simulations for identification of new ECG biomarkers of risk

To date, *in silico* studies of the effects of drug binding to hERG and other channels have largely been focused at the cellular (Mirams *et al*. 2011; Beattie *et al*. 2013; Romero *et al*. 2014) and pseudo‐tissue level (Roden & Viswanathan, 2005; Brennan *et al*. 2009), and these studies have certainly highlight the potential of the approach. The extension of this analysis to the level of the organ is less developed, perhaps because of the computational demands, or simply an acceptance that simpler *in silico* approximations are, on balance, a reasonable compromise given the shortcomings of current molecular and cellular level models that we have highlighted above. However, the feasibility of the approach has been demonstrated in several recent studies. Zemzemi *et al*. reported simulations from human torso and derived a 12 lead ECG, including a basic representation of hERG block (Zemzemi *et al*. 2013). More recently, quantitative analysis of the molecular basis of the T wave based on highly parallelised simulations of many thousands of heartbeats, from hearts with different genetic and molecular backgrounds, highlighted how the subtleties of the T wave shape are related to underlying ion channel conductances (Sadrieh *et al*. 2014). This parallelised approach should prove especially useful when applied to identification of new ECG biomarkers of risk. An efficient approach might be to simulate many thousands of ECGs from hearts with variable tissue structure and genetic backgrounds in the presence of drugs with different kinetic properties and pharmacological profiles to create a library of simulated ECGs that can be screened for ECG biomarkers.

This of course begs the question of what specific properties of the ECG waveform should be considered in the search for these markers of risk? In this regard, there is a growing appreciation that the morphology of the T wave can help paint a much more complete picture of cardiac electrophysiology (Vaglio *et al*. 2008; Johannesen *et al*. 2014; Sadrieh *et al*. 2014; Vicente *et al*. 2015). This idea is supported by clinical data that illustrate how the morphology of the T wave is informative about the range of ion channels blocked by a drug (Vicente *et al*. 2015). More recently research from the Mayo clinic has also demonstrated that specific aspects of T wave morphology – specifically the slope of the descending limb – are correlated with risk of TdP (Sugrue *et al*. 2015). It therefore seems likely that features of the morphology of the T wave on the ECG should be a primary target in our search for new biomarkers. In addition to this, dynamic features of the ECG (such as temporal changes in QT interval and other morphological features of the signal) are a relatively neglected aspect of the ECG that may contribute to better understanding the various arrhythmogenic mechanisms associated with single‐ and multi‐channel drugs (Page *et al*. 2015). The volume of data that can be generated representing a range of pharmacological, genetic and environmental backgrounds using parallelised *in silico* cardiac models presents an opportunity to study these dynamic features in detail and develop them as a complementary facet to T‐wave morphology as ECG biomarkers for drug safety and efficacy. Furthermore, the flexibility of simulation relative to more conventional *in vitro* or *in vivo* assays offers the potential to interrogate a range of rate dependence and complex stimulation protocols, and to correlate the emergence specific ECG parameters with the onset of arrhythmogenic properties (such as cellular EADS) and even the initiation of TdP.

## The future: moving computational cardiology into the clinic

There is still a huge amount of work to be done to achieve the goal embodied in the human Physiome project, i.e. being able to develop individualised *in silico* models, in a realistic timeframe, that can help determine the risk of cardiac arrhythmias in patients. Success will require input from all sectors of the biomedical research enterprise, spanning the academic, industrial and clinical spheres and must be truly multi‐disciplinary. An integrative approach will involve extensive detailed genotyping, comprehensive *in vitro* and *in vivo* phenotyping of multiple systems including electrical, calcium handling, imaging, contractile and metabolic systems, and ultimately high performance computer modelling to integrate the *in vitro* and *in vivo* results to develop quantitative insights into the nature of arrhythmogenic substrates and triggers. This approach, combined with a ‘big data’ analysis of continuous ECG traces will allow the identification of novel ECG biomarkers that have much greater power to discriminate between patients at high risk of proarrhythmic events and those at low risk. Drawing from the experience in the field of aLQTS discussed above, and the gaps in our knowledge that it highlights, we submit that the following specific goals should be pursued as a matter of priority to accelerate the convergence of computational and clinical cardiology:

1. *A model of calcium cycling scalable to tissue and organ level simulation*. Modelling intracellular calcium, even at the single cell level, is currently a bottleneck for *in silico* studies of arrhythmia. Studies using many‐core accelerators such as Intel's Xeon Phi or NVIDIA GPU have mainly focused on increasing the spatial resolution of simulating subcellular calcium down to the nanometer. While these approaches tackle the programming and performance challenges associated with these architectures, our focus should be more on numerical methods to work towards a multiscale model of calcium cycling that can be scaled up to the tissue level, albeit with necessary simplifications.
2. *A consensus model of adrenergic effects on cardiac electrophysiology*. While adrenergic stimulation, either as fight or flight surge, or increased basal tone in the context of ischaemia has been implicated in arrhythmogenesis, this factor is largely absent from models of human ventricular electrophysiology. Particular focus needs to be placed on developing models of the effects of adrenergic stimulation on the major components of repolarisation reserve (*I*_Ks_, *I*_CaL_), as well as intracellular calcium signalling in human cells.
3. *Development of IPSC derived cardiomyocytes for model validation*. Current uptake suggests IPSC derived cardiomyocytes are set to play a major role as a source of human tissue for validation of computer models of cellular electrophysiology and pharmacology as well as providing data to constraint models of particular disease phenotypes. While this goal is outside the immediate computational domain, development of maturation strategies for these cells, such that data can be obtained that is equivalent to adult human tissue, is critical. Furthermore, since industry as well is aggressively pursuing this goal, primarily as part of the CiPA initiative, ongoing collaboration and engagement with industry in this sector is the likely the most productive and efficient approach.
4. *Continuation of efforts to build central repositories and tools for sharing and validation of models*. The CellML language and repository already provide a centralised source for model sharing, while the recently published EP Web Lab allows standardised testing of these models. This resource should be extended to provide a centralised repository for published data, protocols, structural meshes and associated validation datasets such as *in vitro* electrophysiology and clinical ECG data. The Physiome community should explore working groups around facilitating such resources.
5. *Establishment of deep ECG phenotype resources*. Accurate phenotyping of ECG datasets is critical for the iterative improvement of whole heart simulations as well as identification and validation of novel ECG biomarkers of risk identified *in silico*. Such analysis is likely to involve detailed characterisation of ECG wave morphology, as well as the dynamic changes that are associated with emergence of ectopic triggers. These types of analysis will necessitate use of data from Holter and/or implantable loop recorder ECGs to characterise how the heart responds to a wide range of physiological stimuli as well as to capture relatively infrequent, but information rich regions preceding and following ectopic beats.

If such goals can be met, and can be demonstrated to work in a relatively ‘simple’ case such as aLQTS, this will provide an impetus to pursue similar studies in other genetic arrhythmia syndromes (Brugada syndrome, catecholaminergic polymorphic ventricular tachycardia (CPVT), hypertrophic cardiomyopathy (HCM), arrhythmogenic right ventricular cardiomyopathy (ARVC)) and ultimately in the more complex scenario of SCD complicating ischaemic heart disease which involves electrical, calcium, metabolic and structural remodelling.

## Additional information

### Competing interests

None declared.

### Funding

A.P.H. is funded by an Australian Research Council Future Fellowship FT110100075. J.I.V. is funded by a National Health and Medical Research Council fellowship 1019693. G.M. is funded by a joint Wellcome Trust/Royal Society fellowship 101222/Z/13/Z.
